# Cost analysis of negative-pressure wound therapy versus standard treatment of acute conflict-related extremity wounds within a randomized controlled trial

**DOI:** 10.1186/s13017-022-00415-1

**Published:** 2022-02-10

**Authors:** Andreas Älgå, Jenny Löfgren, Rawand Haweizy, Khaldoon Bashaireh, Sidney Wong, Birger C. Forsberg, Johan von Schreeb, Jonas Malmstedt

**Affiliations:** 1 Department of Clinical Science and Education, Södersjukhuset, Karolinska Institutet, Sjukhusbacken 10, 118 83 Stockholm, Sweden; 2 Department of Global Public Health, Karolinska Institutet, 171 77 Stockholm, Sweden; 3 Department of Molecular Medicine and Surgery, Karolinska Institutet, 171 77 Stockholm, Sweden; 4 College of Medicine, Hawler Medical University, 40/0112 Erbil, Iraq; 5 Department of Special Surgery, Jordan University of Science and Technology, 22110 Irbid, Jordan; 6 Medical Faculty, Yarmouk University, Irbid, 21163 Jordan; 7 Médecins Sans Frontières, Operational Centre Amsterdam, Plantage Middenlaan 14, 1018 DD Amsterdam, The Netherlands

**Keywords:** Trauma, Armed conflict, Wounds, Cost analysis

## Abstract

**Background:**

Clinical outcomes after negative-pressure wound therapy (NPWT) and standard treatment of conflict-related extremity wounds are similar. In resource-limited settings, cost affects the choice of treatment. We aimed to estimate treatment-related costs of NPWT in comparison with standard treatment for conflict-related extremity wounds.

**Methods:**

We derived outcome data from a randomized, controlled superiority trial that enrolled adult (≥ 18 years) patients with acute (≤ 72 h) conflict-related extremity wounds at two civilian hospitals in Jordan and Iraq. Primary endpoint was mean treatment-related healthcare costs (adjusted to 2019 US dollars).

**Results:**

Patients were enrolled from June 9, 2015, to October 24, 2018. A total of 165 patients (155 men [93.9%]; 10 women [6.1%]; and median [IQR] age, 28 [21–34] years) were included in the analysis. The cost per patient treated with NPWT was $142 above that of standard treatment. Overall, results were robust in a sensitivity analysis.

**Conclusions:**

With similar clinical outcomes compared to standard care, our results do not support the use of NPWT in routine treatment of conflict-related extremity wounds at civilian hospitals in resource scarce settings.

*Trial registration* NCT02444598.

## Introduction

Extremity wounds and fractures constitute the majority of injuries sustained by civilians during armed conflict [1]. The management of conflict-related injuries is complex and associated with significant challenges [2, 3]. In addition, the resources for healthcare are often limited due to a high burden of disease and injury, and low health system resilience [4]. Negative-pressure wound therapy (NPWT) includes covering the wound and applying a negative pressure and has been used in wound care for more than two decades [5]. In recent years, the technique has been introduced in the treatment of acute injuries sustained in armed conflict despite the weak evidence supporting NPWT as an effective means of promoting wound healing [6]. Data on costs for conflict-related wound treatment are scarce.

In a randomized controlled trial, we compared NPWT and standard treatment of conflict-related extremity wounds [7]. We did not find any superior clinical outcomes for NPWT compared to standard treatment [8]. The proportion who reached the primary endpoint, wound closure by day five, was 49% (*n* = 41/83) in the NPWT group and 60% (*n* = 49/82) in the standard treatment group (risk ratio 0.83, 95% confidence interval 0.62–1.09, *p* = 0.183). The clinical outcomes of the trial have been published in full elsewhere [8]. The aim of the present study was to determine the treatment-related costs of NPWT compared to the costs of standard treatment.

## Methods

This is a health economic evaluation of a randomized controlled trial comparing outcomes from conflict-related extremity wound treatment using NPWT and standard treatment (NCT02444598) [7]. The study findings are reported in accordance with the Consolidated Health Economic Evaluation Reporting Standards (CHEERS) guidelines [9].

### Trial procedures

The design and clinical outcomes of the randomized controlled trial have been described in detail elsewhere [7, 8]. Briefly, 165 adult (≥ 18 years) patients with acute (≤ 72 h) conflict-related extremity wounds were enrolled from June 9, 2015, to October 24, 2018, at two civilian hospitals in Jordan and Iraq. Participants were randomly assigned to NPWT (*n* = 83), involving a commercial NPWT device with a continuous negative pressure of 125 mm Hg, or standard treatment (*n* = 82), involving wound dressings with non-adhesive sterile gauze covered with a bandage. Dressings were changed in the operating theatre every three to five days, in accordance with the International Committee of the Red Cross war surgery protocol [10]. The primary outcome was wound closure by day five. Data on wound closure were collected at each dressing change, at hospital discharge, and at days 14 and 30 following the day of randomization. Wound closure was defined as closure by suture, flap, or split-thickness skin graft. A coprimary endpoint, net clinical benefit, was used, defined as a composite of wound closure by day five, and freedom from any bleeding, wound infection, sepsis, or amputation of an index limb. Health outcome data from both study sites were used for the present study.

### Cost analysis

The cost analysis was undertaken from the perspective of the healthcare provider [11]. Costs were either related to surgeries or to the care given on the ward and included the following items: medicines and materials, staff costs, overhead costs, and capital costs (Table 1). A surgical procedure was defined as any intervention that occurred in the operating theatre, including wound dressing changes. Wound dressing changes were not performed on the ward. The cost per surgical procedure was calculated based on the total number of surgeries per year. Costs for postoperative care on the ward were calculated as cost per 24 h, based on the yearly costs for all admitted patients divided by the yearly number of patient-days on the ward.

**Table 1:** Items included in the cost analysis

Item	Definition	Source of information	Cost calculation method
Medicines and materials			
Operating theatre	Mean cost for intraoperative medicines and materials	Participating surgeons and nurses	No. of items per surgery × price per item
Postoperative care	Mean cost for postoperative medicines and materials	Participating surgeons and nurses	Ward annual usage/total annual patient hospital time (days)
Staff costs			
Operating theatre	Mean cost for one surgeon, one anesthesiologist, one anesthesiologist assistant, and one operating theatre nurse	Hospital pay roll	Operating theatre yearly salary costs/number of surgeries per year
Postoperative care	Mean staff cost for one day on the ward	Hospital pay roll	Annual salary of standard set of staff at the ward/total annual patient hospital time (days)
Overhead costs			
Operating theatre	Overhead costs proportional to operating theatre space in relation to whole hospital	Measurement of building, hospital end-of-year report	Theatre proportion of total hospital space × annual expenditure/annual no. of operations
Postoperative care	Overhead costs proportional to ward space in relation to whole hospital	Measurement of building, hospital end-of-year report	Ward proportion of total hospital space × annual expenditure/total annual patient hospital time (days)
Capital costs			
Operating theatre	Potential income if the area of the theatre had been used for land lease	Measurement of building, land leasing prices in Erbil city	Area used for theatre × yearly cost of leasing land in Erbil city with size equal to theatre area/annual no. of operations
Postoperative care	Potential income if the area of the ward had been used for land lease	Measurement of building, land leasing prices in Erbil city	Area used for ward × yearly cost of leasing land in Erbil city with size equal to ward area/total annual patient hospital time (days)
Equipment	Cost of equipment per surgery, major equipment depreciated over 10 years, NPWT pumps over 3 years	Hospital staff	Depreciation calculated as cost/no. of years each item is depreciated over
Other			
Food	Meals for patients in hospital	Cost of equivalent meals purchased at local restaurant	Cost of meals per day
Hygiene items, washing, and cleaning	Costs for hygiene items, washing, and cleaning proportional to space in relation to whole hospital	Hospital end-of-year report	Proportion of total hospital space × annual expenditure/annual no. of operations OR total annual patient hospital time (days)

NPWT, negative-pressure wound therapy

The Iraqi site, where information was readily available to the study team, provided cost data. During 2017, the Iraqi hospital provided a total of 6169 patient days and 2210 surgeries, exclusively for patients with acute conflict-related injuries. Costs for the treatment of chronic wounds (defined as non-closure within 30 days) were not included in the analysis. All NPWT equipment was bought for the purpose of this study. Local costs were converted to average 2019 US dollars.

#### Medicines and materials

Mean cost for single-use items (e.g., dressing materials and NPWT sponges) used for dressing changes (NPWT and standard treatment) as well as mean cost for medicines was calculated based on the amount used during the surgical procedures. Data were provided by the participating surgeons and nurses.

#### Staff costs

Standard staffing in the operating theatre was one surgeon, one anesthesiologist, one anesthesiologist assistant, and one perioperative nurse. Standard staffing on the ward was one nurse per four patient beds. Data were obtained from the hospital pay roll.

#### Overhead costs

Overhead costs included costs for water, electricity, transportation, and administration. Data were extracted from the hospital’s end-of-year report. The overhead costs applied to the treatment of the study patients were based on the patients’ proportional use of hospital space (operating theatre and the ward).

#### Capital costs

Capital costs were incurred for hospital buildings (operating theatre and the ward) and for equipment used for the surgical procedures and on the ward. For this study, the value of the hospital buildings was estimated based on the income if the area occupied by the buildings had been used for land lease. The capital cost of the operation theatre and the ward was estimated by multiplying the capital cost of each building by the proportion of the total surface area allocated to each of these units. To calculate the capital cost per surgery, the capital cost of the operating theatre was divided by the annual number of surgeries. The capital cost per day spent at the hospital was calculated by dividing the capital cost of the ward by the total number of patient days on the ward. Major equipment, such as the operating table and autoclave, was depreciated over 10 years. NPWT pumps were depreciated over three years.

#### Other

Other costs included costs for food, hygiene items, washing, cleaning, and waste management. Data were extracted from the hospital’s end-of-year report.

### Sensitivity analysis

To assess the robustness of the findings, a sensitivity analysis was performed. The surgical productivity level was modified by − 20% and by + 20%, and staff costs were modified by − 50% and by + 50%. To assess the effects of a rural hospital setting, rental costs were modified by − 50%. The cost for NPWT pumps used in this study was lower than standard costs in high-resource settings. Thus, the capital cost for the NPWT pumps was modified by + 100%.

### Statistical analysis

We analyzed data using R version 3.5.0 software [12].

## Results

**Fig. 1:**
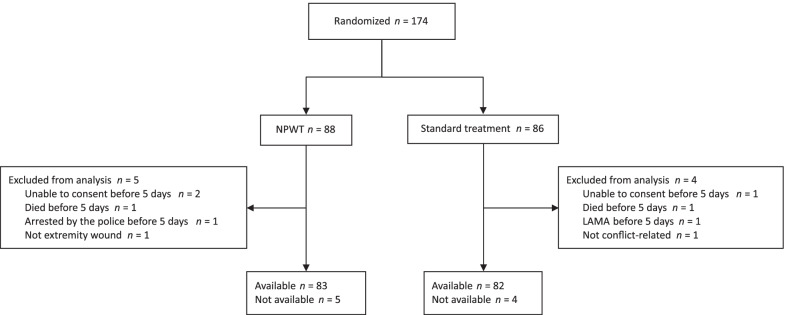
Trial profile. NPWT, negative-pressure wound therapy; LAMA, left against medical advice

A total of 165 patients (155 men [93.9%]; 10 women [6.1%]; and median [IQR] age, 28 [21–34] years) were included in the study. Randomization and analysis are depicted in Fig. 1. The groups were well balanced in baseline characteristics.

### Costs

**Fig. 2:**
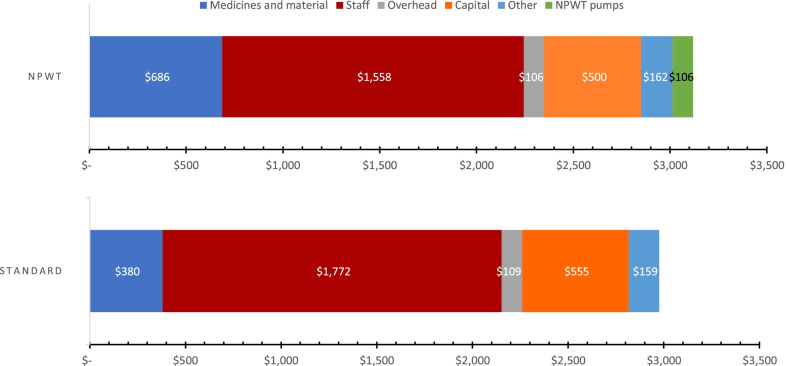
Mean costs per treated patient. Currency data are in 2019 US dollars. NPWT, negative-pressure wound therapy

The cost per surgery was $329 in the NPWT group and $250 in the standard treatment group. The cost per day spent at the hospital was $116 and $109 in the NPWT and standard treatment groups, respectively. The mean patient cost for the full hospital period was $3118 in the NPWT group and $2976 in the standard treatment group (Table 2). Consequently, the use of NPWT was associated with an additional $142 (5%) per treated patient compared to standard treatment (Fig. 2).

**Table 2:** Mean patient costs using negative-pressure wound therapy (NPWT) and standard treatment

Item	NPWT, costs, $		Standard treatment, costs, $
Medicines and materials			
Intraoperative medicines (incl. anesthesia)	12.2		14.5
Intraoperative materials (incl. anesthesia equipment and dressing materials)	349.3		65.9
Postoperative medicines	90.7		83.8
Postoperative materials	234.3		216.5
Staff costs			
Staff costs in operating theatre (1 surgeon, 1 anesthesiologist, 1 anesthesiologist assistant, 1 perioperative nurse)	668.6		950.4
Staff costs for postoperative care (1 nurse per 4 patient beds)	889.9		822.4
Overhead costs			
Share of operating theatre, autoclaving area, water, electricity, transport, administration and records office	21.1		30.1
Share of ward, laboratory, water, electricity, transport, administration and records office	84.6		78.2
Capital costs			
Share of operating theatre and autoclaving area	142.6		202.7
Equipment (anesthesia machine, operating table, operating theatre light, autoclave)	44.7		63.6
Share of ward and accessory buildings	313.4		288.7
NPWT pumps	106.0		0
Other			
Hygiene items, washing, and cleaning, operating theatre	20.4		29.0
Hygiene items, washing, and cleaning, ward	44.7		41.2
Food (3 meals per day)	96.4		89.0
Total	3117.8		2975.9

Currency data are in 2019 US dollars

### Sensitivity analysis

Table 3 shows a sensitivity analysis of mean patient costs using NPWT and standard treatment. While some changes did occur with respect to the mean difference in cost, patients allocated to NPWT had consistently higher costs.

**Table 3:** Sensitivity analyses of mean patient costs using negative-pressure wound therapy (NPWT) and standard treatment

	NPWT	Standard treatment	Incremental cost (%)
Baseline	3118	2976	142 (5%)
Sensitivity analyses			
Productivity level			
80%	3756	3678	78 (2%)
120%	2692	2508	184 (7%)
Staff costs			
50%	2321	2073	248 (12%)
150%	3915	3879	36 (1%)
Hospital location, rural (rental costs 50%)	2889	2729	160 (6%)
Capital cost, NPWT pumps (200%)	3224	2976	248 (8%)

Currency data are in 2019 US dollars

## Discussion

In this health economic evaluation of a pragmatic, randomized, controlled superiority trial in patients with acute conflict-related extremity wounds, the overall cost for treatment was higher in the NPWT group compared to the standard treatment group. Our previous results showed no significant differences in clinical outcomes for NPWT compared to standard treatment in this setting [8]. The present study adds information on treatment costs for civilians with conflict-related wounds when managed using NPWT and standard treatment.

Health economic evaluation is essential when considering implementation of new treatment methods, particularly in resource-limited settings [13]. Despite NPWT being a costly mode of treatment, it has been widely implemented without robust evidence of effectiveness nor of cost-effectiveness [14]. Of the few published randomized controlled trials on NPWT for traumatic injuries, only one included an economic evaluation [6]. Petrou et al. assessed NPWT in 460 patients with open lower limb injuries in a high-income setting and could not show any cost-effectiveness benefit for NPWT compared to standard treatment [15]. Healthcare in all contexts entails choices about resource allocation, and interventions should be guided by public health considerations. This requires maintained quality of care, guaranteed effectiveness of treatment, and justification of costs. Introducing treatment methods that increase costs, without clinical benefit, is not justifiable, especially not in settings where resources are scarce.

Limitations to this study include the use of assumptions to calculate costs, and the use of proxies in the absence of cost information, which might have introduced bias. In addition, there is a risk of facility bias as cost data could only be retrieved from one of the two study hospitals. However, the absence of information often represents a challenge to health economic evaluations, and assumptions are therefore commonly used. Costs were calculated using the same methods for both treatment groups, and therefore, we believe the cost comparison is reliable. As costs depend on hospital setting, the cost differences between NPWT and standard treatment will vary. However, the sensitivity analysis indicates robustness of our results and describes a variation of costs in several different scenarios, which is advantageous for policy and decision-making. The real-world setting at acute surgery hospitals that do not perform elective surgery may allow for generalization to similar populations of injured civilians. The pragmatic study design increases the external validity, which generally is a concern with health economic evaluations based on randomized trials [16].

To our knowledge, this is the first health economic evaluation of NPWT for traumatic wounds carried out in a resource-limited setting. Although we found no support for the use of NPWT, the technique may serve purposes not assessed in this study, such as improving the quality of life by affecting patients’ discomfort, wound-associated pain, and sleep quality. In addition, the role for NPWT in the treatment of chronic wounds and in patients treated with open abdomen technique still needs to be defined [17–19]. These areas are all in need of further investigation.

## Conclusions

Among patients with acute conflict-related extremity wounds treated at two civilian hospitals, NPWT did not decrease costs nor improve health outcomes, as compared to standard treatment. Wide-scale introduction of NPWT for the management of conflict-related extremity injuries cannot be recommended.

## Data Availability

Our ethics approval limits us to the publication of results at the aggregate level only, precluding us from publishing individual-level patient data. Data underlying the results are available for researchers who meet the criteria for access to confidential data. Data will be available with publication and for 10 years subsequently. Proposals should be directed to andreas.alga@ki.se.

## Abbreviations

CHEERS: Consolidated Health Economic Evaluation Reporting Standards
IQR: Interquartile range
NPWT: Negative-pressure wound therapy
